# *Aster tataricus* extract and its active compounds display a broad spectrum of antiviral activity in vitro and in vivo

**DOI:** 10.1186/s13020-025-01167-1

**Published:** 2025-07-15

**Authors:** Nuwan Gamage, Ji-Won Cha, Ji-Soo Jeong, Yebin Seong, Kiramage Chathuranga, Asela Weerawardhana, Jin Yeul Ma, Tae-Won Kim, Jong-Soo Lee

**Affiliations:** 1 Department of Microbiology, College of Veterinary Medicine, Chungnam National University, Daejeon, Republic of Korea; 2 Department of Pharmacology, College of Veterinary Medicine, Chungnam National University, Daejeon, Republic of Korea; 3 Korean Medicine Application Center, Korea Institute of Oriental Medicine, Daegu, Republic of Korea

**Keywords:** *Aster tataricus*, Antiviral effect, Active compounds, Herbal medicine

## Abstract

**Background:**

*Aster tataricus*, a perennial terrestrial herb with a rich history of use in traditional medicine, is renowned for its therapeutic properties. However, despite the widespread use of *Aster tataricus*, its antiviral efficacy and mode of action against viruses have not yet been studied. Here, we demonstrated that *Aster tataricus* extract (ATE) has antiviral effects and an underlying mechanism of action both in vitro and in vivo.

**Methods:**

Antiviral effect of ATE was assessed against Influenza A virus (PR8), Newcastle Disease Virus (NDV), and Herpes Simplex Virus-1 (HSV) in RAW264.7 cells. Mechanism was explored by analyzing the induction of antiviral immune responses, including type-I interferon (IFN) signaling and cytokine secretion. In vivo, BALB/c mice were treated with ATE prior to infection with lethal influenza A subtypes, A/PR/8/34 (H1N1), A/Aquatic bird/Korea/W81/2005 (H5N2), and A/Chicken/Korea/116/2004 (H9N2). Survival rates, viral titers, and lung pathology were measured. High-performance liquid chromatography (HPLC) was used to identify active compounds in ATE, and their antiviral effects were further investigated.

**Results:**

An effective dose of ATE significantly inhibited influenza A virus (PR8), NDV, and HSV replication in RAW264.7 cells. Mechanistically, we found that ATE induced an antiviral state, which includes upregulation of type-I interferon signaling and secretion of IFNs and pro-inflammatory cytokines in RAW264.7 cells. In vivo, ATE treatment showed increased survival due to reduced viral titers and less severe pathological changes in the lung, and the observed prophylactic effects were associated with increased secretion of IL-6, IFN-γ, and IFN-β in bronchoalveolar lavage fluid. Based on the reported information and HPLC analysis, quercetin, kaempferol, and ferulic acid were identified as active compounds in the aqueous fraction, and an effective dose of each compound exhibited antiviral effects similar to ATE against influenza viruses.

**Conclusions:**

These findings suggest that ATE and its active compounds act as immunomodulators and may be potential candidates as a source of promising natural antivirals for animals and humans.

**Supplementary Information:**

The online version contains supplementary material available at 10.1186/s13020-025-01167-1.

## Introduction

Numerous viruses contribute to the prevalence of malignant diseases on a global scale, leading to significant loss of life and economic repercussions [1]. Notably, influenza A viruses cause recurring seasonal outbreaks and have resulted in three pandemics over the twentieth century [2]. The inherent reservoir of influenza A virus, which has evolved to infect a diverse array of animals, is aquatic bird species, but the virus can also infect dogs, horses, swine, domestic poultry, and other animals. Thus, there are a lot of host species through which zoonotic viruses can be transmitted to humans [3]. Indeed, Influenza virus infects around 10% of the global population on an annual basis, posing a significant health risk and leading to an estimated 250,000 deaths [4]. Beyond influenza viruses, other viral pathogens like herpes simplex (HSV) and Newcastle disease (NDV) pose significant challenges to public health and agriculture. HSV is a DNA virus belonging to the *Herpesviridae* family. HSV-1 affects approximately 67% of the global population under age 50 and causes recurrent infections due to its ability to establish latent infections [5, 6]. NDV is a highly infectious paramyxovirus that primarily affects avian species. It causes severe disease in susceptible birds, particularly chickens, where mortality rates can exceed 50% of infected flocks. While primarily an avian pathogen, NDV can occasionally cause mild conjunctivitis and flu-like symptoms in humans who handle infected birds. [7]. The success of viruses as pathogens stems from their ability to mutate quickly, leading to the emergence of new variants that can evade vaccine-induced immunity and circulate among the population. Consequently, a single immunization strategy is insufficient for long-term disease control [8]. In addition, various prophylactic and therapeutic drugs, as well as vaccines, have been created to combat and treat viral diseases, including Influenza virus. The effectiveness of these treatments is limited due to the emergence of new variants, rapid development of antiviral resistance, and associated side effects [9, 10]. To develop more effective methods for preventing viral diseases, research has focused on identifying natural products with broad-spectrum antiviral activity that are inexpensive and rapidly applied with common antiviral protocols.

The innate immune system functions as the frontline protection against viral infections, with the type I interferon (IFN) response being pivotal in this protective mechanism [11]. Following viral detection by pattern recognition receptors (PRRs), signaling pathways activate transcription factors, including interferon regulatory factors (IRFs) and nuclear factor kappa B (NF-κB), resulting in the production and secretion of type I IFNs [12–14]. These cytokines bind to the IFN receptor (IFNAR) on neighboring cells, triggering JAK-STAT signaling pathway and promoting the expression of interferon-stimulated genes (ISGs), which foster an antiviral environment [15]. This mechanism is important for controlling viral replication and spreading during the early stages of infection, making it a promising target for immunomodulatory antiviral strategies.

Throughout history, societies have employed natural herbal medicines to treat human diseases. As stated by the World Health Organization (WHO), about 20,000 different plant species are employed for medicinal uses. Products derived from herbal medicines, specifically natural compounds, show efficacy against influenza and other viruses. Traditional herbal remedies are garnering increased interest owing to their accessibility, affordability, safety, promising efficacy, and environmentally friendly nature [16–19]. Many people from diverse nations rely on traditional herbal remedies, recognizing their significant contribution to public health and overall well-being. Utilizing natural products, whether in the form of purified individual active compounds or standardized botanical extracts, offers boundless prospects for developing novel antiviral medications characterized by high efficacy, low toxicity, and minimal side effects.

Tatarian aster, scientifically named *Aster tataricus*, is a plant that belongs to the Asteraceae family and is indigenous to areas including Siberia, Korea, Japan, and various parts of eastern Asia [20]. This perennial terrestrial herb, which boasts violet-blue flowers with a conspicuous yellow center, has been utilized for over 2,000 years in traditional Chinese medicine to treat throat infections, pneumonia, snake bites, tonsillitis, and bronchial infections [21]. The plant has yielded a diverse array of bioactive compounds, encompassing various chemical classes such as terpenoids, amides, saponins, flavonoids, lignans, and sterols [22–24]. Also, the anti-inflammatory [25], anti-cancer [26–28], hepato-protective [29] anti-microbial, anti-asthma, and anti-diabetic [30] properties of *Aster tataricus*, as well as its immunosuppressive activity [31], have been studied. However, the antiviral effects of *Aster tataricus* and its identified active compounds have not been investigated in depth. Despite its diverse biological properties, the immuno-modulatory potential and underlying mechanism(s) of action of *Aster tataricus* are still not well understood based on current scientific evidence.

Here, we comprehensively explored the effects of total aqueous *Aster tataricus* extract (ATE) against a wide array of viruses both in vitro and in vivo. Moreover, we verified its potential to modulate the mechanisms that govern innate immune responses. Furthermore, we employed high-performance liquid chromatography (HPLC), pinpointing the active compounds within the aqueous fraction.

## Materials and methods

### Preparation of *Aster tataricus* extract

*Aster tataricus* plant materials were purchased in dried form from the Jaecheon Oriental Herbal Market (Republic of Korea). Identity and quality were authenticated by Professor Ki-Hwan Bae from the College of Pharmacy at Chungnam National University. The aqueous extract was prepared at the Herbal Medicine Improvement Research Center, Korea Institute of Oriental Medicine (Daejeon, Republic of Korea). Briefly, extraction was performed by immersing 100 g of dried plant materials in 1 L of distilled water (DW) and then subjected to extraction by heating at 105 °C for 2.5 h. The resulting extract was filtered using a 0.45 μm (Millex) filter and then centrifuged at 12,000 rpm for 15 min. Following pH adjustment to 7.0, a Second filtration was performed using a 0.22 μm syringe filter to ensure sterility. The sample was then diluted to 1.0 mg/ml and preserved at −20 °C for future experiments.

### Cells and viruses

Murine macrophages (RAW264.7 ATCC^®^ TIFB-71), Vero cells (ATCC^®^ CCL-81^™^), and MDCK cells (ATCC^®^ CCL-34, NBL-2) were cultured in Dulbecco’s Modified Eagle’s Medium (DMEM) (Cytiva) enriched with 10% fetal bovine serum (FBS) and 1% antibiotic/antimycotic (AA) solution (Gibco, NY, USA) at 37 °C with 5% CO_2_. GFP-tagged Influenza A (A/Puerto Rico/8/34(H1N1) (PR8-GFP), Newcastle Disease Virus (NDV-GFP) and challenge viruses of influenza A subtypes A/Aquatic bird/Korea/W81/2005(H5N2), A/PR/8/34 (H1N1), and A/Chicken/Korea/116/2004 (H9N2) were amplified in the 10-day-old chicken embryos. The Herpes Simplex Virus-1 tagged with Green Fluorescent Protein (HSV-GFP) was amplified in Vero cells.

### Removal of endotoxin contaminants

Endotoxin removal was performed by filtering the samples through Acrodisc units equipped with a 0.2 µm Mustang E membrane (MSTG25E3; Pall Corporation, NY, USA), maintaining the flow rate at 1 ml/min. Filtered eluates were analyzed for any remaining endotoxins using Toxin Sensor^™^ Chromogenic LAL Endotoxin Assay Kit (Gene script, Piscataway, NJ, USA). Endotoxin levels were measured using a standard calibration curve, following the manufacturer’s instructions.

### Assessment of 50% cytotoxic (CC_50_) and effective (EC_50_) concentration

To evaluate CC_50_, RAW264.7 cells were prepared (2.5 × 10^4^ cells/well) in duplicate within a 96-well plate. After a 12 h incubation period, cells were subjected to a series of increasing ATE concentrations (0, 0.98, 1.95, 3.91, 7.81, 15.63, 31.25, 62.5, 125, 250, and 500 µg/ml) and kept under incubation for 24 h. Cell viability was determined using (3-(4,5-dimethylthiazole-2-yl)−2,5-diphenyltetrazolium bromide (MTT) assay [32, 33]. Briefly, 10 µl of MTT reagent was added to each well. 100 µl of DMSO was added after a 5 h incubation at 37 °C. Colorimetric alterations were quantified. Absorbance was recorded at a wavelength of 595 nm. 50% cytotoxic concentration (CC₅₀) was calculated from the absorbance values using a standard statistical approach based on a non-linear regression model. To determine the EC₅₀, 1 × 10^5^ cells/well in 24-well plates were added with two-fold dilutions of ATE at varying concentrations (µg/ml) in DMEM. Cells were washed once with PBS at 12 h post-treatment (hpt), followed by infection with PR8-GFP (MOI = 1), HSV-GFP (MOI = 3), and NDV-GFP (MOI = 3) in DMEM containing 1% FBS. At 2 h post-infection (hpi) media changed, and fluorescence intensity was quantified at 24 hpi (GloMax multi-detection system, Promega). Graphs were developed based on the dilutions (log values of concentration) and percentage of GFP expression. EC₅₀ values were determined using extract concentration required to achieve a 50% reduction in GFP expression.

### Antiviral assays in ATE pretreated RAW264.7 cells

A GFP-based virus replication inhibition assay was carried out following a previously documented procedure, with minor modifications [34]. RAW264.7 cells were grown in 12-well culture plates (2.5 × 10^5^ cells/well). Following a 12 h incubation, cells were introduced with 1,000 U/ml rmIFN-β (positive control; Sigma, St. Louis, USA) or DMEM containing 10 μg/ml ATE (10 μl/ml or 1% v/v). DMEM alone served as an untreated and virus-only control. At 12 hpt, cells were subjected to virus infection (PR8-GFP; MOI = 1, NDV-GFP; MOI = 3, and HSV-GFP; MOI = 3) in a medium of 500 µl of DMEM containing 1% FBS. Culture media were substituted at 2 hpi, followed by a 24 h incubation period. GFP expression, viral titration, and cell survival were assessed at 12 and 24 hpi.

### Quantification of NDV-GFP mRNA and viral titers in RAW264.7 cells

Total mRNA was extracted to evaluate the expression level of NDV-GFP mRNA [35]. RAW264.7 cells were seeded in 12-well tissue culture plates. Following a 12 h incubation period, cells were exposed to either 10 μg/ml ATE or DMEM alone. Cells were subsequently infected with NDV-GFP (MOI = 3) at 12 hpt, with sample collection at 12 and 24 hpi. RNeasy Mini Kit (Qiagen, Hilden, Germany) was used to isolate total RNA, converted to cDNA, and subjected to PCR amplification [36] using specific primers (Supplementary Table 1). PCR products were equally loaded onto 1.5% ethidium bromide agarose gels and visualized (GelDoc Imaging System, Bio-Rad). Band intensities of the matrix (M) gene were standardized against GAPDH.

### Virus titration from ATE-treated cells

Viral titers were assessed using a standardized plaque assay in Vero cells. Briefly, virus-infected cells were harvested at 12 and 24 hpi from each group and underwent five cycles of freezing and thawing. The resulting cell suspensions were resuspended in 500 μl of PBS, followed by a series of dilutions, before being utilized for cell infection. Vero cells were maintained until they reached 70–80% confluency, after which they were inoculated with the virus. At 2 hpi, the virus inoculum was discarded and replaced by agar at a concentration of 0.45 g per 20 ml of DW. The overlaid cells were further incubated for an additional 36–48 h at 37 °C for plaque development. Viral titers were calculated based on the number of plaque-forming units (PFU) and corresponding dilution factors.

### Time of addition assay for ATE

To assess the pre-treatment effect of ATE, cells in 12-well culture plates were treated with 10 μg/ml ATE (equivalent to 10 μl/ml or 1% v/v) at designated time intervals (12, 8, 4, and 2 h) prior to viral infection. PR8-GFP (MOI = 1) was prepared in 500 µl of DMEM containing 1% FBS and added to each well. Following 2 hpi, medium was refreshed with DMEM and incubated for 24 h. GFP fluorescence was observed using microscopy. After imaging, absorbance was measured. ATE post-treatment effect was examined where cells were seeded and allowed to incubate for 12 h, ATE (10 µg/ml) was administered at 2, 4, 8, and 12 h after exposure to the virus. Incubation continued for 24 h, and fluorescence was visualized and quantified.

### Virucidal activity, virus attachment, and entry assay

For the virucidal assay, ATE (10 µg/ml) was mixed with PR8-GFP (MOI = 1) in a 500 µl DMEM and incubated for 20 min at 37 °C. Incubated Virus and ATE mixture were then introduced to RAW264.7 cells (2.5 × 10^5^ cells/well). At 2 hpi inoculum was replaced with DMEM. GFP absorbance was measured after 24 h of incubation. To investigate ATE-mediated inhibition of virus-cell attachment, cells were incubated with the virus-ATE mixture (10 μg/ml ATE with PR8-GFP (MOI = 1) at 4 °C for a duration of 2 h to facilitate infection, medium was substituted with DMEM, and the cells underwent for 24 h incubation at 37 °C . GFP fluorescence was measured. For entry assay, RAW264.7 cells were inoculated with PR8-GFP (MOI = 1) for a duration of 2 h at 4°C. After washing the cells, ATE 10 μg/ml was introduced and incubated at 37°C . GFP expression was quantified 24 hpt.

### Enzyme-linked immunosorbent assay (ELISA)

Cells were administered either 1000 U/ml rmIFN-β, 10 μg/ml ATE, or DMEM alone as a control. After 12 and 24 h of incubation, culture supernatants were collected for cytokine analysis. Concentrations of murine interleukin-6 (IL-6) and tumor necrosis factor-alpha (TNF-α) were measured using ELISA kits from BD Biosciences (USA), while murine IFN-β levels were quantified using a commercial ELISA kit from CUSABIO (Houston, USA), following the manufacturers’ protocols.

### Assessment of ATE-induced protein phosphorylation by immunoblot analysis

In order to analyze protein expression, RAW264.7 cells were treated with LPS (100 ng/ml), which served as a positive control, or 10 μg/ml ATE. DMEM alone was employed as a negative control. Cells were collected at 0, 8, 12, and 24 hpt and lysed. Cell lysates were subjected to SDS-PAGE and subsequent immunoblotting with specific antibodies. Antibodies used as follows: anti-TBK1 (Cell Signaling: 3504S), anti-phosphoTBK1 (Cell Signaling: 5483S), anti-IRF3 (Abcam: 25950), (Ser396) anti-phospho-IRF3 (Cell Signaling: 4947), anti-STAT1 (Cell Signaling: 9175), (Y701) anti-phospho-STAT1 (Cell Signaling: 9167), anti-NF-κB P65 (Cell Signaling: 4764S), anti-phospho-NF-κB-P65 (Cell Signaling: 3031S) anti-IkBα (Cell Signaling: 9242S), anti-phospho-IkBα (Cell Signaling: 2859S) and anti-β-actin (Santa Cruz: SC47778).

### Oral inoculation of ATE and influenza viral challenge in BALB/c mice

BALB/c mice (5 week old) from Hanil Laboratory Animal Center (Jeonju, Korea), used to conduct in vivo experiments, to conduct influenza virus challenge experiments, fifty-four (54) mice were clustered into 3 main groups (n = 18), each including 3 subgroups, The group treated with PBS served as a negative control (n = 6), group treated with rmIFN-β acted as a positive control (n = 6) and ATE inoculated group (n = 6). Another 18 mice were grouped into 3 subgroups for lung virus titration: control (n = 6), IFN-treated (n = 6), and ATE-treated (n = 6). At 3 dpi and 5 dpi, lung tissues were collected from each group of mice, with three samples taken at each time point (3 dpi; n = 3 and 5 dpi; n = 3). At 5 dpi, the left lung lobe was promptly preserved in 10% formaldehyde for histopathological examination during the sample collection. ATE was orally administered to the mice at 0.1 mg/ml ATE (20 μg per head) two times, specifically on days 1, 3, 5, and 7 prior to infection. The control group received an oral dose of 200 μl of PBS. Mice were subjected to an intranasal infection with H1N1, H5N2, or H9N2, each delivered at a dose equivalent to five times the 50% minimum lethal dose (MLD50), using 20 μl PBS (Supplementary Table 2). The monitoring of body weight and survival continued until 13 dpi. Mice that experienced a body weight reduction exceeding 25% were identified as having reached the experimental endpoint and were euthanized humanely. The experiments involving treatment and challenge were carried out in a BSL-2 laboratory facility with the necessary approvals.

### Determination of viral burden and histological changes in lungs

Assessment of lung virus titers performed using a 50% tissue culture infectious dose (TCID_50_) assay [37]. Briefly, homogenized lung tissues in PBS supplemented with antibiotic and antimycotic solution were subsequently centrifuged (12,000 rpm; 15 min) to eliminate cellular debris. The samples, which had been serially diluted, were incubated for 1 h with MDCK cells in 96-well culture plates. Overlay medium supplemented with L-1-tosylamide-2-phenylethyl chloromethyl ketone trypsin [(TPCK), Sigma-Aldrich] was replaced with DMEM, and cells underwent 3–4 days of incubation, after which cytopathic effects (CPE) were examined under a microscope. Viral titers were quantified using the Reed and Muench method and presented as log₁₀ TCID₅₀ per lung tissue [38]. Tissues intended for histopathological examination were immediately preserved with 10% formalin. Briefly, the central part of the tissue sections with a thickness of 4–6 mm was excised, mounted on slides, and then stained with hematoxylin and eosin (H&E). Histopathological changes were assessed under light microscopy [39, 40].

### Evaluation of cytokines and RT-PCR in vivo

Mice (n = 4) were given an oral dose of 0.1 mg/ml ATE, with a total volume of 200 μl, administered twice on days 1, 3, 5, and 7. Control mice were given PBS. Serum and bronchoalveolar lavage fluid (BALF), were obtained. Blood was drawn from the retro-orbital plexus and centrifuged to obtain serum. BALF was obtained by flushing 1 ml of Hank’s Balanced Salt Solution (HBSS, Gibco) into the lung through the trachea three times, subjecting it to centrifugation (10,000 × g, 10 min, 4 °C), and cytokine levels were analyzed using ELISA. Lung tissue samples for RT-PCR were obtained and stored at −70 °C until use [41]. RT-PCR primers are detailed in Supplementary Table 1.

### HPLC analysis

The liquid chromatography-ultraviolet (LC-UV) analysis was performed on an Agilent 1200 Series high-performance liquid chromatography (HPLC) (Agilent Technologies Co., USA). ZORBAX Eclipse XDB-C18 (2.6 × 150 mm, I.D.−3.5 μm) (Agilent Technologies Co., USA) column was utilized for chromatographic analysis. The mobile phase consisted of 0.1% formic acid (Solvent A) and acetonitrile (Solvent B) in the gradient mode as follows: 0–1 min 3% B; 1–2 min 3–15% B; 2–13 min 15–50% B; 13–20 min 50–100% B; 20–23 min 100% B; 23–23.5 min 100–3% B; 23.5–27.5 min 3% B at a flow rate of 0.25 ml/min at 40 °C. Injection volume was 5 µl, and the UV detector was set at 360 nm. Quercetin, Kaempferol, and Ferulic acid were quantified in ATE. These three compounds were selected for further analysis based on the literature [20, 42].

### Determination of antiviral characteristics of HPLC-selected chemical compounds

Antiviral properties and the capacity to stimulate cytokine production were further assessed using three chemicals identified from HPLC in immune cells. GFP absorbance (PR8-GFP) in RAW264.7 cells, virus titration, and cytokine secretion were observed following the procedures detailed in the previous sections.

### Statistical analysis

All graphs and statistical analyses were generated using GraphPad Prism; version 9. Results are expressed as mean ± SD from at least three independent experiments. Statistical comparisons between control and treatment groups at each time point were conducted using an unpaired t-test. *P < 0.05, **P < 0.01 or *** P < 0.001 were considered statistically significant.

## Results

### Impact of ATE on the time of addition, virucidal activity, attachment, and entry of the PR8-GFP (H1N1) influenza virus

**Fig. 1:**
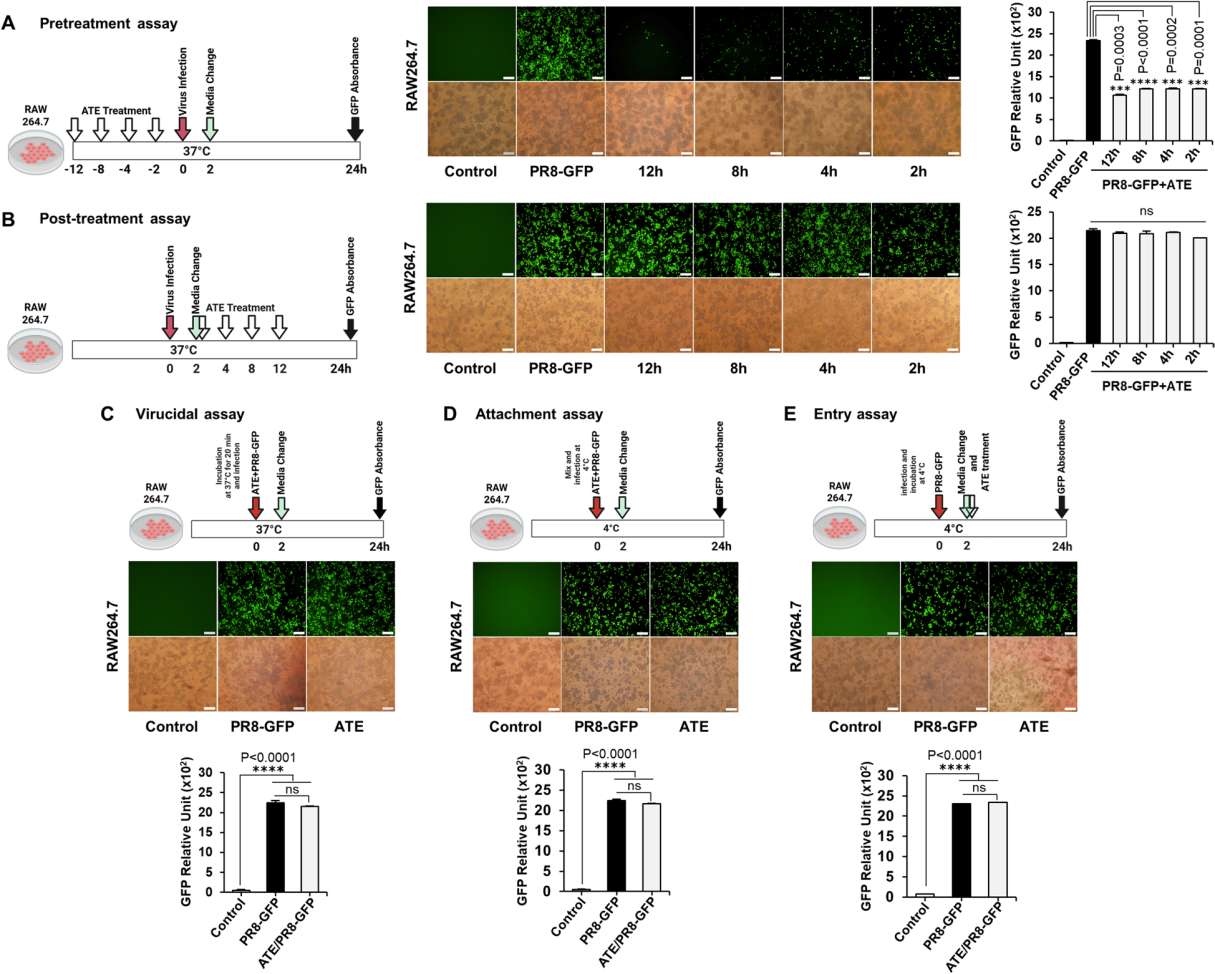
Antiviral activity of ATE using time-of-addition, virucidal, virus attachment, and entry assays. **A** Pretreatment: ATE (10 μg/ml) was applied at various time points prior to PR8-GFP infection. **B** Post-treatment: ATE was added 2, 4, 8, and 12 h after viral infection. **C** Virucidal assay: ATE was mixed with PR8-GFP before infection. **D** Attachment: Virus-ATE mixtures were incubated with cells at 4 °C. **E** Virus entry inhibition: After incubating the virus with cells at 4 °C for a duration of 2 h, ATE was subsequently administered. Viral replication was assessed using GFP fluorescence and absorbance. Each data point reflects mean ± SD from three separate experiments. Scale bar: 50 μm. (*P < 0.05, **P < 0.01, ***P < 0.001)

The timing of administration plays a pivotal role in determining the antiviral efficacy of natural compounds. Prior to assessment of antiviral activity, endotoxins were removed from the ATE preparation such that endotoxicity was acceptable for in vivo utilization according to Food and Drug Administration Agency (FDA; USA guidelines) recommendations (Fig. S1A). As an initial inquiry into the influence of ATE on viral replication, we first conducted a time of addition assay using RAW264.7 cells. To evaluate the utility of ATE as a pre-treatment candidate, it was added to cells at different intervals prior to virus infection (Fig. 1A). Notably, introduction of ATE at 12, 8, 4, and 2 h before the onset of infection demonstrated a marked ability to suppress viral activity; however, when we examined its effect on H1N1 suppression at 2, 4, 8, and 12 h post-infection (hpi) (Fig. 1B), we found none. We noted that delaying addition of ATE to infected cells markedly reduced its ability to suppress viral activity. Furthermore, investigations into the effects of ATE on virucidal activity (Fig. 1C), viral attachment (Fig. 1D), and entry (Fig. 1E) suggested that the compound had no notable impact on suppressing viral entry or initiation of replication. Therefore, subsequent efforts were directed toward exploring the antiviral effects of ATE when applied before rather than after virus infection.

### Antiviral potential of ATE in RAW264.7 cells and its ability to inhibit replication of different viruses

**Fig. 2:**
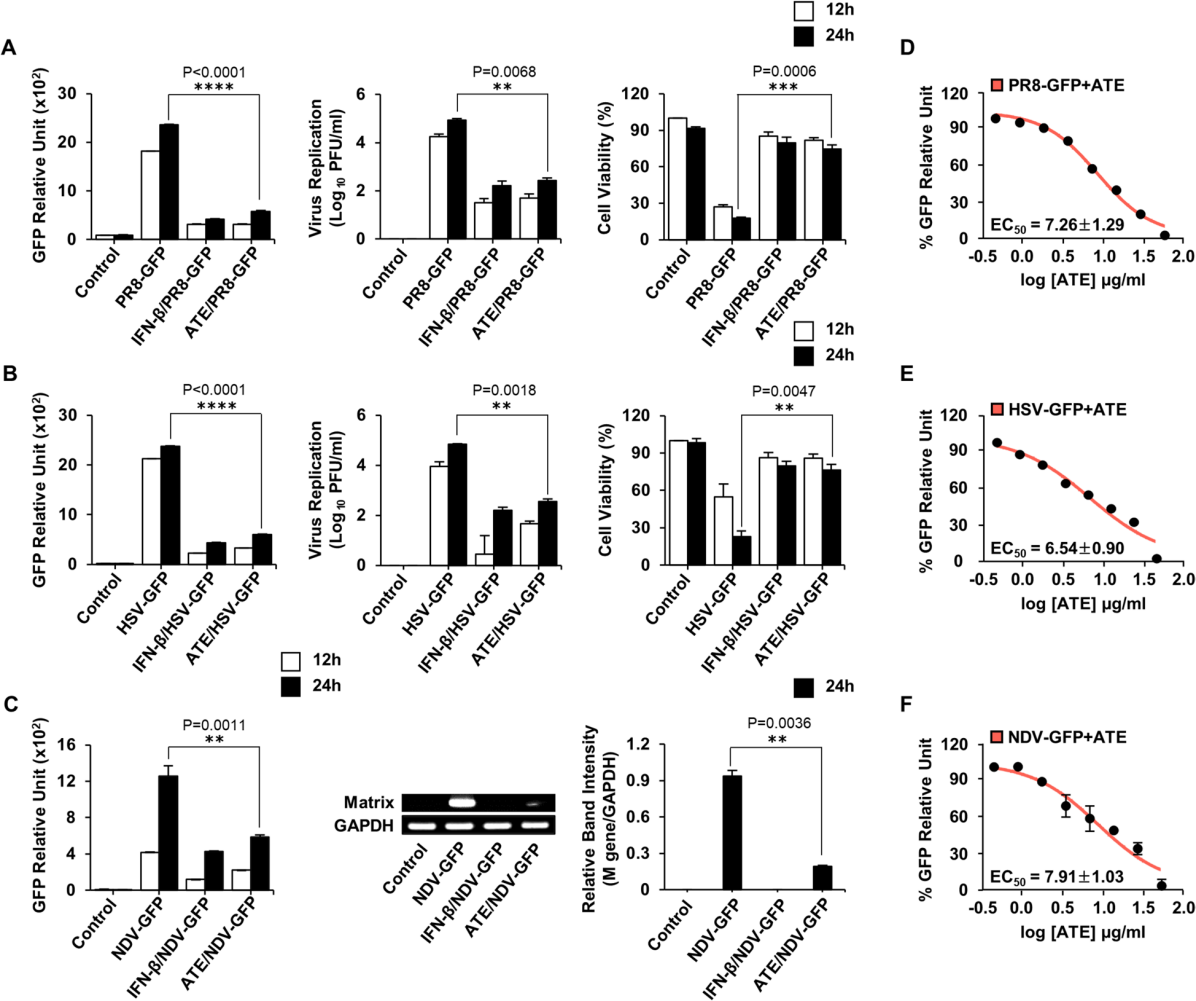
Antiviral effects of ATE against PR8-GFP, HSV-GFP, and NDV-GFP in RAW264.7 cells. Cells were pretreated for 12 h with media alone, ATE (10 μg/ml), or rmIFN-β (1,000 U/ml), followed by **A** PR8-GFP **B** HSV-GFP or **C** NDV-GFP infection. Fluorescence was measured to evaluate viral replication. Viral titers were assessed using plaque assays for PR8-GFP and HSV-GFP, while NDV M-mRNA expression was analyzed via RT-PCR and normalized to GAPDH. PCR products were visualized. Relative band intensities (RBI) were calculated as M-mRNA/GAPDH ratio. MTT assay was employed to measure cell viability, and the findings were expressed as a percentage compared to the control. **D**–**F** EC₅₀ was evaluated by treating cells with increasing concentrations of ATE prior to infection. GFP intensity was measured at 24 hpi, and EC₅₀ values were calculated. Data displayed as the mean ± SD. (*P < 0.05, P < 0.01, *P < 0.001)

To determine whether the observed antiviral activity of ATE can also inhibit the replication of a diverse range of viruses, antiviral assays were performed in vitro against various GFP-expressing viruses. RAW264.7 cells treated with ATE 10 μg/ml [10 μl/ml or 1% v/v] prior to infection by PR8-GFP, demonstrated a notable decrease in GFP expression when compared with untreated cells (Fig. 2A). This was also the case for cells infected with HSV-GFP (Fig. 2B) and NDV-GFP (Fig. 2C). A marked decrease in virus replication was evident in ATE-treated cells, consistent with observed viral titers. ATE reduced the titer of PR8-GFP by twofold, and that of HSV-GFP by 1.9-fold. Crucially, ≥ 70% of cells treated with ATE remained viable for up to 24 hpi. In contrast, untreated cells showed markedly higher levels of cell death after virus infection. The effects of ATE were similar to those of IFN-β, which was used as a positive control. RT-PCR performed to measure expression of mRNA encoding the M-gene, which drives replication of NDV, was lower in ATE-treated cells than in untreated cells (Fig. 2C, right panel). Results indicate that ATE possesses inhibitory activity against all three viruses in RAW264.7 cells.

### Identification of the EC_50_ and CC_50_ of ATE in vitro

The EC_50_ is defined as the concentration of extract that results in a 50% inhibition in virus titer, whereas the concentration that results in 50% cell viability is defined as the CC_50_. Table 1 and Fig. S1B show that the CC_50_ of ATE for RAW264.7 cells is 134.0 ± 1.08 µg/ml. A modified GFP assay utilizing RAW264.7 cells was developed to measure in vitro EC₅₀ values of ATE against various viruses [35, 43]. Given that we utilized GFP-tagged viruses exclusively, a 50% reduction in GFP expression was deemed equivalent to a 50% reduction in the virus titer. The EC_50_ of ATE for PR8-GFP, HSV-GFP, and NDV-GFP were 7.26 ± 1.29 μg/ml, 6.54 ± 0.90 μg/ml, and 7.91 ± 1.03 μg/ml, respectively (Table 1 and Fig. 2D–F). Based on these data, ATE is relatively nontoxic, and a concentration of 10 μg/ml was considered appropriate and effective for use in the anti-viral assays.

**Table 1:** Determination of EC_50_ and CC_50_ of ATE in RAW264.7 cells

	RAW264.7			
	PR8-GFP	HSV-GFP	NDV-GFP	CC_50_ ± SD^b^ (μg/ml)
EC_50_ ± SD^a^ (μg/ml)	7.26 ± 1.29	6.54 ± 0.90	7.91 ± 1.03	134.0 ± 1.08
SI (CC_50_/EC_50_)	17.71	20.44	18.66	

^a^Effective concentration for 50% reduction in GFP expression

^b^Cytotoxic concentration causing 50% cell death

### Pre-treatment with ATE inhibits replication of both RNA and DNA viruses in a concentration-dependent manner

Pre-treatment of RAW264.7 cells with ATE demonstrated potent and dose-dependent antiviral effects against both PR8-GFP and HSV-GFP in vitro. When cells were pre-treated with increasing concentrations of ATE (5, 10, and 20 µg/ml), we observed progressively reduced GFP fluorescence intensity compared to virus controls, indicating significant inhibition of viral replication. This antiviral effect was comparable to that observed with IFN-β (10000 U/ml), which served as a positive control. Quantitative analysis confirmed that ATE significantly decreased GFP expression in a concentration-dependent manner for both viruses. The highest concentration (20 µg/ml) showed the most pronounced suppression against PR8-GFP (Fig. S2A) and HSV-GFP (Fig. S2C). To validate these findings, we measured virus titers using cells and supernatants of the same treatment groups. Consistent with the GFP expression data, ATE-treated cells displayed significant and dose-dependent reductions in viral titer for both PR8-GFP (Fig. S2B) and HSV-GFP (Fig. S2D). These findings reveal that ATE orchestrates a robust, concentration-dependent suppression of viral replication, with efficacy that progressively intensifies as dosage increases, suggesting that ATE acts through host-directed mechanisms to confer potent antiviral protection against diverse range of viral infections.

### ATE induces secretion of IFN-β, pro-inflammatory cytokines, and expression of antiviral-related genes, as well as activating type I IFN signaling

**Fig. 3:**
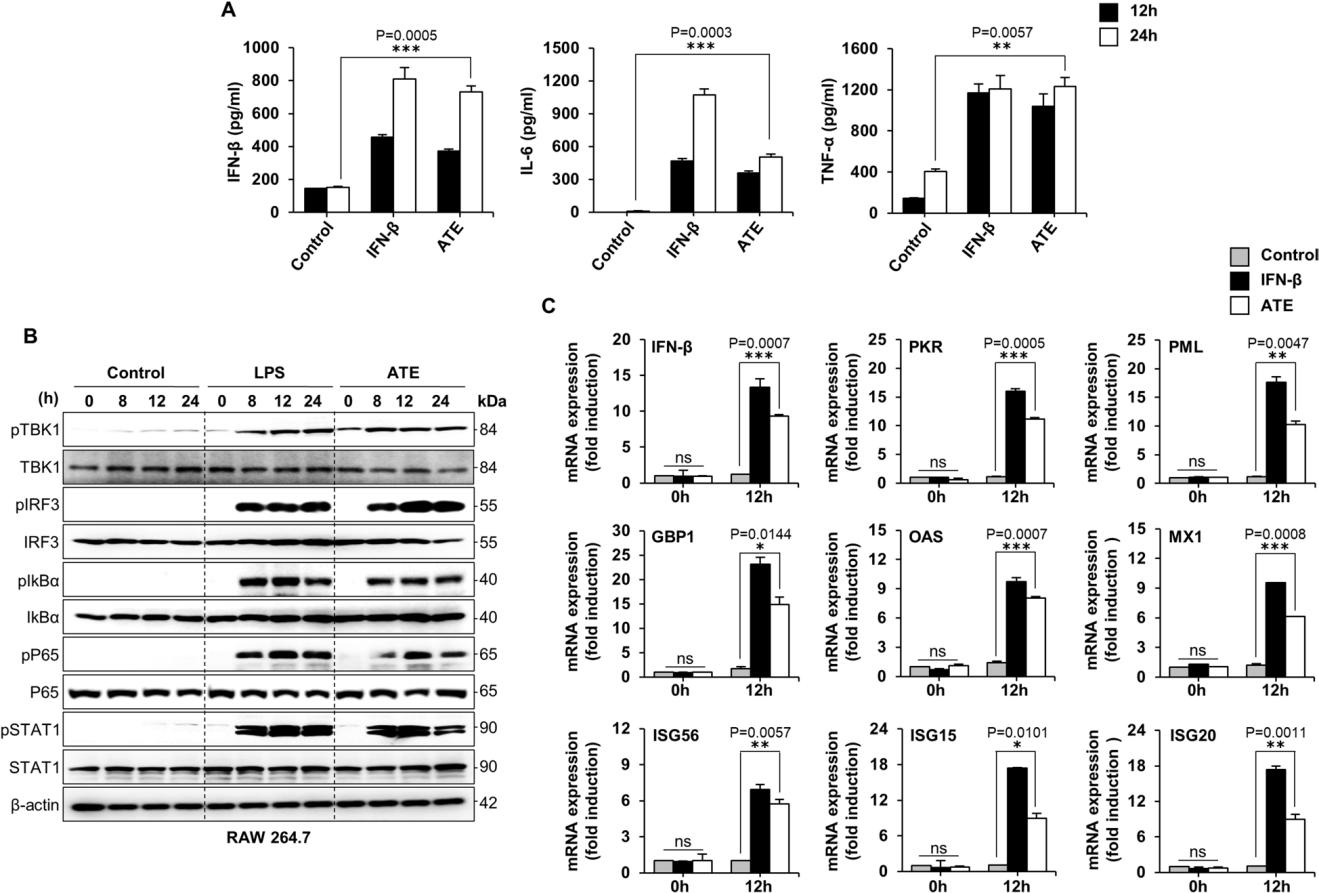
ATE induces cytokine production, antiviral gene expression, and phosphorylation of type I IFN signaling. **A** RAW264.7 cells were exposed to either media alone, ATE at 10 μg/ml, or rmIFN-β at 1,000 U/ml, and levels of IFN-β, IL-6, and TNF-α were quantified by ELISA at 12 and 24 hpt. **B** Protein phosphorylation related to type I IFN and NF-κB pathways was assessed by immunoblotting. **C** Expression of IFN-stimulated genes was measured by RT-PCR at 0 and 12 hpt, with GAPDH used for normalization. The data are shown as the mean ± SD. (*P < 0.05, **P < 0.01, ***P < 0.001)

Given the antiviral activity exhibited by ATE, we decided to examine its potential effects on IFN signaling pathway. To explore this possibility, we quantified IFN-β and other pro-inflammatory cytokines secretion in the supernatant of ATE-treated RAW264.7 cells (Fig. 3A). ATE (10 μg/ml) led to an increased secretion of IFN-β, IL-6, and TNF-α at both 12 and 24 h post-treatment (hpt). These results suggest that ATE stimulates secretion of IFNs and other pro-inflammatory cytokines, fostering an antiviral environment that inhibits viral replication. Based on these data, we hypothesized that there might be a link between ATE treatment and activation of IFN and NF-κB-induced signaling pathways. To investigate this, we measured phosphorylation of IFN-related signaling molecules and NF-κB activation-related molecules after exposure to ATE. Immunoblot analyses of cell lysates prepared from ATE-treated (10 μg/ml) RAW264.7 cells revealed that ATE increased phosphorylation of key signaling molecules within the IFN-I and NF-κB pathways, such as TBK1, IRF3, STAT1, IκBα, and P65 significantly (Fig. 3B). In particular, IRF3 phosphorylation, a crucial indicator of IFN signal transduction, was initiated at 8 hpt and remained elevated over time. Further, activated P65, a core element involved in NF-κB transcription, triggered marked secretion of pro-inflammatory cytokines. Moreover, an augmented level of STAT1 phosphorylation suggested transcriptional activation of ISGs, which are crucial for governing responses to viral infection. ATE-induced phosphorylation of these molecules was comparable with that observed after treatment with LPS, a stimulator of TLR4 [44] and a potent activator of multiple innate immune signaling pathways, including NFκB (pIĸBα, pP65) and IRF3 (pTBK1, pIRF3) [45, 46]. Next, we asked whether ATE activates antiviral genes and ISGs in RAW264.7 cells. As verified by real-time PCR, ATE upregulated the expression of mRNAs encoding diverse antiviral and ISGs at 12 hpt. The observed level of transcription was comparable to those induced by IFN-β, which served as a positive control (Fig. 3C). Collectively, these results imply that ATE induces secretion of interferons and pro-inflammatory cytokines by activating signaling molecules involving type I IFN and NF-κB signaling cascades.

### Orally administered ATE protects BALB/c mice against lethal challenges from various influenza A subtypes

**Fig. 4:**
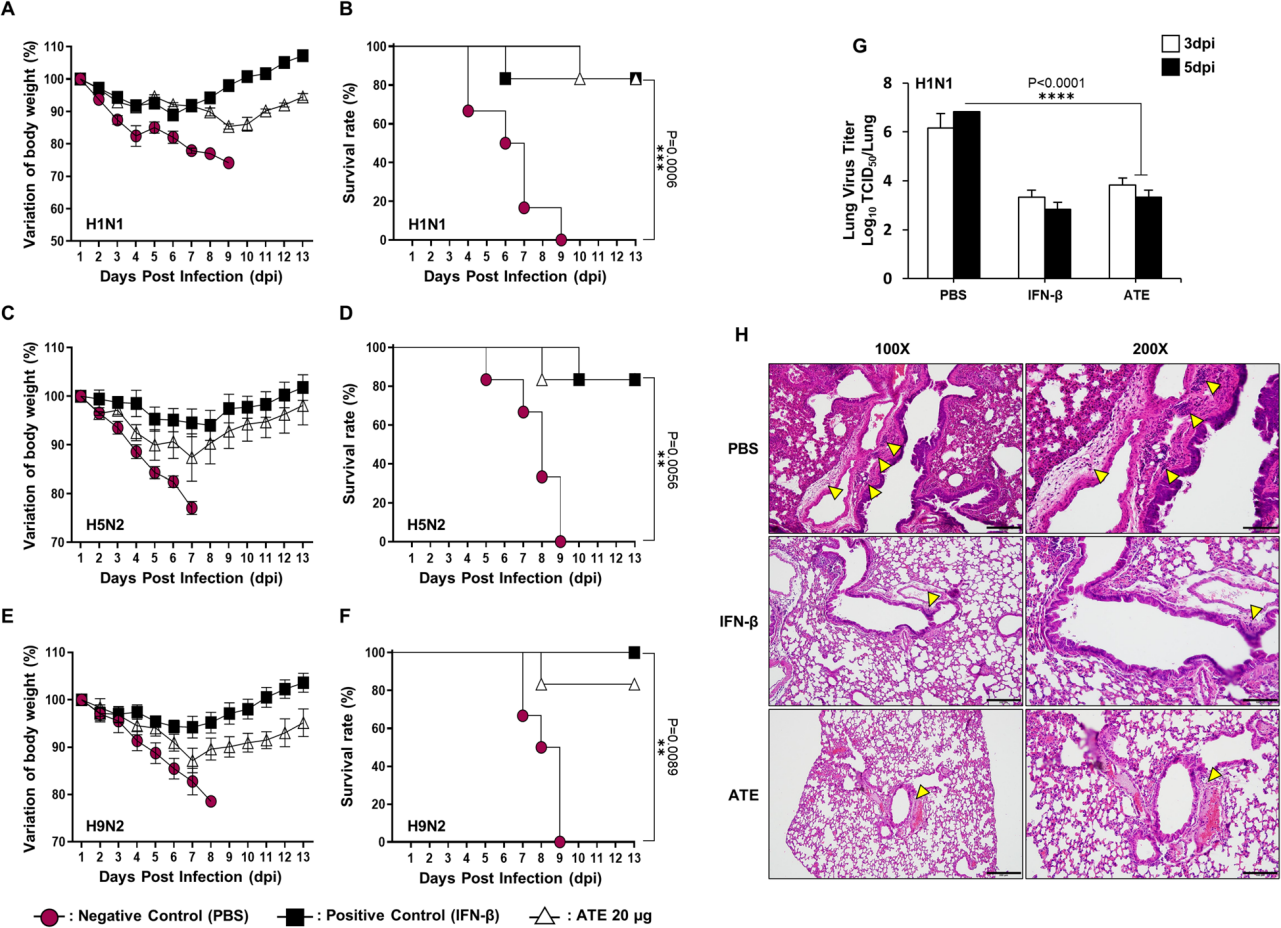
Oral ATE administration protects BALB/c mice against lethal challenge with Influenza A virus subtypes. Mice received oral ATE (20 μg/head, 0.1 mg/ml in 200 μl) prior to infection with 5 MLD₅₀ of each virus. Survival rates and body weight changes were measured up to 13 dpi. **A**, **B** H1N1, **C**, **D** H5N2, or **E**, **F** H9N2. **G** Using TCID₅₀, the lung viral loads of mice infected with H1N1 were evaluated at 3 and 5 dpi. **H** Lung histopathology at 5 dpi was examined by hematoxylin and eosin (H&E) staining; arrows highlight regions where inflammatory cells have infiltrated. Data represents mean ± SD. Images were captured at 100 × and 200 × magnifications (scale bars: 200 μm and 100 μm, respectively). *P < 0.05, **P < 0.01, or ***P < 0.001

Next, to investigate the in vivo efficacy of ATE against influenza viruses, BALB/c mice were gavaged with ATE at a dose of 20 μg per mouse for 7 days. Selection of a minimum effective dose of 20 μg/head was based on our previous in vivo experimental results using other herbal extracts. After oral inoculation, mice were intranasally challenged with 5 MLD_50_ of A/PR/8/34 (H1N1), A/Aquatic bird/Korea/W81/2005 (H5N2), or A/Chicken/Korea/116/2004 (H9N2) viruses. Following infection, control group of mice experienced severe illnesses, characterized by significant loss of body weight. At around 3–4 dpi, a majority of untreated mice exhibited serious clinical manifestations of respiratory disease, such as labored respiration and respiratory distress. All viruses tested caused complete mortality in the control group by 9 dpi. Interestingly, Mice that received ATE treatment exhibited ≤ 20% body weight loss between 5–7 dpi, followed by gradual recovery from 8–9 dpi, and all mice returned to their normal state by 12–13 dpi (Fig. 4A, C, E). ATE treatment achieved a survival rate exceeding 80% in mice challenged with H1N1 (Fig. 4B), H5N2 (Fig. 4D), and H9N2 (Fig. 4F). Apart from mild weight loss, no evident clinical signs were observed in the surviving mice.

To better understand the protective effect of ATE, we selected the H1N1 infection model for additional analysis of viral replication and lung pathology. The influenza virus is transmitted primarily through aerosols and replicates most effectively in the lungs [47]. Therefore, we collected lung tissues from the H1N1-infected and control groups. Notably, lungs obtained from untreated mice displayed evidence of efficient H1N1 replication, with viral titers of 6.16 and 6.83 log TCID_50_/Lung at 3 and 5 dpi, respectively. By comparison, the viral burden in the ATE-treated groups was significantly lower, at 3.83 and 3.33 log TCID_50_/Lung at 3 and 5 dpi, respectively (Fig. 4G). Histopathological examination of lung sections revealed marked alterations between H1N1-infected mice receiving PBS and those treated with ATE. For example, control mice harbored pronounced pathological alterations such as perivascular edema and infiltration of the alveoli by inflammatory cells. ATE treatment mitigated these changes, leading to reduced perivascular edema and inflammatory cell infiltration into pulmonary tissue (Fig. 4H). These findings suggest that administering ATE orally reduces viral replication in the lungs, which in turn enhances the survival rate of mice infected with deadly doses of the influenza virus. Consequently, ATE provides extensive protection against infections from diverse influenza A subtypes.

### Oral administration of ATE increases levels of IFNs and cytokines in serum and Bronchoalveolar Lavage Fluid (BALF) and promotes the expression of cytokine mRNAs in lung homogenates of BALB/c mice

**Fig. 5:**
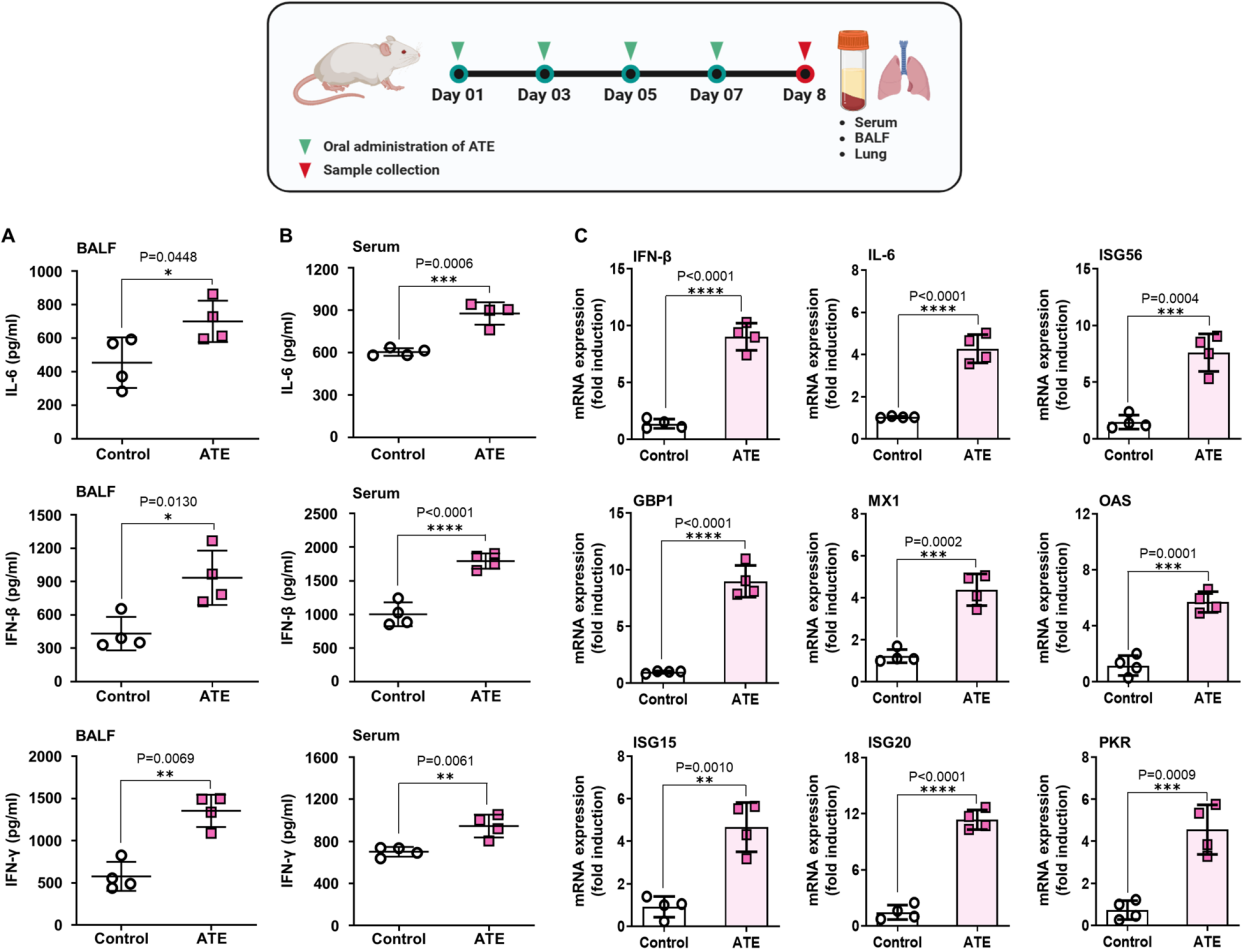
Oral ATE administration enhances cytokine and antiviral gene expression in mice. BALB/c mice (n = 4) received ATE (20 μg/head) or PBS on days 1, 3, 5, and 7. At 24 h after the final dose, **A** BALF and **B** serum were collected for ELISA detection of IFN-β, IL-6, and IFN-γ. **C** Lung tissues were analyzed for ISG expression using RT-PCR. Results are shown as mean ± SD. *P < 0.05, **P < 0.01, and ***P < 0.001

So far, our experiments show that oral administration of ATE protects mice against influenza infection. Therefore, we decided to examine the underlying mechanism(s) through which ATE confers protection against viral infection in mice. First, we assessed the levels of IL-6, IFN-β, and IFN-γ in serum and BALF. As expected, mice administered with ATE exhibited significantly higher levels of pro-inflammatory and antiviral cytokines in serum and BALF than the PBS-treated group (Fig. 5A, B). These results reinforce the idea that ATE orchestrates an antiviral response in BALB/c mice by inducing expression of pro-inflammatory cytokines and IFNs, which may enhance the antiviral state in BALB/c mice significantly, thereby playing a key role in hindering viral replication. This prompted us to investigate lung homogenates of mice to assess the induction of a range of antiviral genes and ISGs in response to ATE treatment. Transcription of various ISGs, pro-inflammatory cytokines, and IFNs was substantially increased in homogenates from ATE-treated mice than in PBS-treated mice. Furthermore, ATE increased transcription of ISG15, ISG20, and ISG56 by 4.5-fold, eightfold, and 7.5-fold respectively, and of IFN-β, PKR, OAS, and MX1 by 4.5-fold, 5.6-fold, 4.5-fold, and fourfold, respectively (Fig. 5C). These data collectively imply that ATE elicits systemic immune responses that confer protection against viral infection in mice.

### Identification of the active compounds responsible for the antiviral effects of ATE

**Fig. 6:**
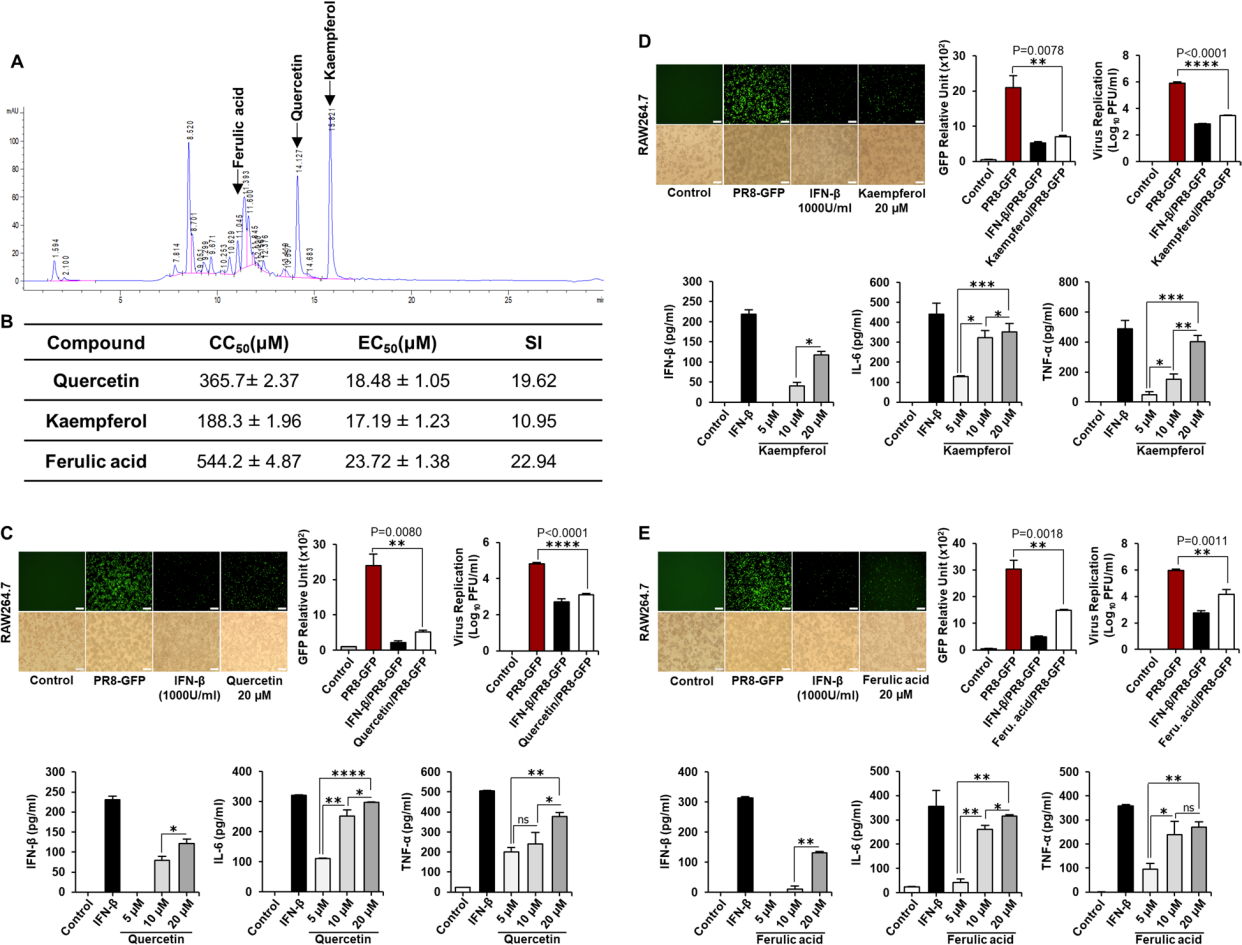
HPLC analysis of ATE and antiviral activity of Quercetin, Kaempferol, and Ferulic acid **A** Chemical components of ATE were identified using reverse-phase HPLC. **B** CC₅₀, EC₅₀, and SI values of quercetin, kaempferol, and ferulic acid were determined in RAW264.7 cells. **C**–**E** Cells were pretreated with 20 μm of each compound or rmIFN-β (1000 U/ml) before PR8-GFP infection (MOI = 1). At 24 hpi, GFP expression, viral titers, and cytokine levels (IFN-β, IL-6, and TNF-α) were measured. Values represent the mean ± SD of independent experiments. Scale bar: 50 μm. Statistical significance; *P < 0.05, **P < 0.01, and ***P < 0.001

HPLC analysis was performed to pinpoint the potential antiviral compounds existing in ATE. Based on the reported information and HPLC analysis, quercetin, kaempferol, and ferulic acid were identified as potential bioactive constituents exhibiting antiviral activity against influenza virus. These compounds were detected at relevant wavelengths and retention times. (Fig. 6A and Fig. S3). The CC_50_ and EC_50_ of quercetin, kaempferol, and ferulic acid were measured in vitro assays using RAW264.7 cells. Among identified phytochemicals, kaempferol exhibited the highest cytotoxicity (CC_50_ = 188.3 µM). The CC_50_ values for quercetin and ferulic acid were 365.7 µM and 544.2 µM, respectively. The lowest antiviral effects were induced by ferulic acid (23.72 µM), while quercetin and kaempferol yielded comparable outcomes (18.48 µM and 17.19 µM, respectively) (Fig. 6B and Fig. S4). The comparatively high selectivity index of these phytochemicals against influenza viruses underscores their potential as effective antiviral agents under biological conditions. Building on this, the antiviral effects of kaempferol, quercetin, and ferulic acid against PR8-GFP influenza virus were examined when administered prior to infection. The minimum effective dose (20 µM) for all selected compounds was based on our preliminary investigations assessing the efficacy of quercetin, kaempferol, and ferulic acid. Interestingly, each chemical effectively suppressed virus replication (Fig. 6C–E). The chemical-treated cells displayed significantly lower expression of GFP compared to untreated controls. The observed GFP data paralleled the viral titer; the chemicals reduced the titer of PR8-GFP significantly 24 hpi. Furthermore, each chemical had a dose-dependent effect on cytokine secretion, with a minimum effective dose of 20 µM, by RAW264.7 cells (Fig. 6C–E). These findings strongly suggest that kaempferol, quercetin, and ferulic acid, major constituents of ATE, induce an antiviral state in cells, thereby inhibiting virus replication.

## Discussion

Influenza viruses present a considerable public health risk, a phenomenon closely linked to their ability to mutate and their propensity for recombination [48–50]. Influenza virus strains mutate continuously, and the genomic segments have the potential for reassortment, leading to emergence of new virus subtypes [51, 52]. This inherent variability of viruses poses significant challenges to development of vaccines and drugs. These challenges include resistance, economic considerations, and side effects. Therefore, research has shifted its focus toward exploring multiple potential antiviral drugs that exert effective antiviral activity [53]. Plants with their extensive evolutionary history of gaining resistance to viruses make them an attractive, promising source of antiviral compounds. More precisely, some plant extracts and their associated chemicals exhibit efficacy against influenza viruses [54, 55]. No study has reported the antiviral effects of ATE, despite evidence of its many medicinal uses to treat cancer, asthma, diabetes, and bacterial and fungal infections.

Here, we extensively demonstrated the antiviral activity of ATE both in vivo and in vitro. Initially, we found that pre-treating RAW264.7 murine macrophages with ATE reduced replication of influenza (PR8-GFP), NDV-GFP, and HSV-GFP markedly. Second, we determined the CC_50_ and EC_50_ values of ATE. Third, we showed that ATE induces antiviral effects by stimulating innate immune responses and producing an antiviral state in RAW246.7 cells. Furthermore, BALB/c mice orally administered ATE were protected from infection by influenza viruses. Finally, to identify the critical components within ATE, we used reversed-phase HPLC. Among several candidates, three major chemical constituents were identified, all of which play pivotal roles in the antiviral efficacy of ATE.

Administration of highly concentrated medicinal herbs can have cytotoxic effects, which poses a challenge when conducting clinical studies [56]. Initially, we found that ATE exhibited no notable cytotoxic effects on RAW264.7 cells. The CC_50_ was several magnitudes higher than the EC_50_. The calculated selective indices for multiple viruses indicated a marked safety margin for the extract, supporting its potential use for therapeutic or prophylactic purposes. Importantly, the results indicated that ATE exhibited a substantial pre-treatment effect, whereas it did not demonstrate significant efficacy as a post-treatment candidate. In vitro studies revealed that the potency of pre-treatment is comparable with that of IFN-β, a key marker and modulator of the body's antiviral defense mechanisms [57]. This suggests that ATE holds promise as a potential pre-treatment candidate for influenza.

In anticipation of viral infection, host cells promptly recognize the pathogen and initiate innate immune responses, such as release of pro-inflammatory cytokines and type I IFNs [58]. Initiating an antiviral state during the initial phase of viral attack is pivotal for controlling viral transmission and disease progression [59]. Our results suggest that ATE induces an antiviral state in macrophages by increasing secretion of interferons and pro-inflammatory cytokines. ATE triggered secretion of IFN-β, IL-6, and TNF-α, as well as inducing transcription of ISGs in vitro. Activation of IFN-I cascade is a fundamental immune defense response against invading viral pathogens [60, 61]. ATE activated the IFN-I and NF-κB signaling cascades by increasing phosphorylation of crucial signaling molecules within these pathways.

Oral administration of ATE not only enhanced the survival of mice subjected to lethal challenge with H1N1, H5N2, and H9N2 but also facilitated rapid body weight recovery. Additionally, surviving mice had lower virus titers in the lungs from influenza challenge model, a finding consistent with the in vitro data. Moreover, histopathological analysis of lung tissues demonstrated that ATE ameliorated perivascular edema and reduced infiltration of lung tissue by inflammatory cells (Fig. 4H). We also assessed immune status by measuring cytokine levels in serum and BALF from ATE-inoculated BALB/c mice. We detected significant increases in IL-6, IFN-β, and IFN-γ levels in both serum and BALF. Prior studies suggest that increased levels of serum IL-6 or IFN-β are associated with an antiviral state, which plays a crucial role in inhibiting virus replication [62, 63]. Indeed, oral administration of ATE upregulated ISGs expression, pro-inflammatory cytokines, and IFN mRNA markedly in BALB/c mice, highlighting its potential to boost systemic immunity and protect against influenza.

The protection provided by oral inoculation of ATE is most likely due to a combination of active compounds. ATE comprises an extensive array of active compounds, including fatty acids, esters [64], amides [65], peptides [66–68], flavonoids [69], phenolic acids, saponins [70], and terpenoids [64, 70]. It is therefore crucial to identify the specific chemicals responsible for its antiviral effects. To compare with known chemicals, we performed HPLC and identified ferulic acid, kaempferol, and quercetin as three major compounds responsible for initiating antiviral defense mechanisms. Several in vitro studies have demonstrated anti-influenza A activity of quercetin and its derivatives [71–76]. Studies conducted in vivo in animal models of influenza virus infection showed that quercetin and its derivatives considerably decreased mortality rates and delayed the onset and progression of pulmonary lesions [77–79]. Park et al. highlighted the antiviral potential of the herbal extract against influenza, with a specific focus on the presence of kaempferol in the extract [80]. Kaempferol has also been reported to exhibit antiviral activity against the pandemic H1N1 influenza virus in vitro, additionally, it has shown the ability to significantly mitigate influenza symptoms in mice [81]. Over the past decades, numerous extracts rich in bioactive phenolic compounds, including ferulic acid as a main or secondary component, have been assessed for their antiviral activity [82–84]. For instance, ferulic acid is identified as one of the major phenolic compounds in the extract of *Hamulus lupulus* that was found active against various strains of the influenza virus [83]. Hariono et al. [85] also report inhibitory activity of ferulic acid isolated from *Garcinia mangostana* against the neuraminidase of H1N1. We extend this previous knowledge by conducting in vitro antiviral activity tests using all three compounds. The data demonstrated that pre-treatment of RAW264.7 cells with each of the three components inhibited replication of A (H1N1) virus. The three compounds exhibited comparable EC_50_ and CC_50_ values, resulting in dose-dependent increases in IFN-β, IL-6, and TNF-α secretion (Fig. 5). Thus, the data suggests that the antiviral effects of ATE stem primarily from the combined impact of all three of these active compounds in the extract. While we successfully identified quercetin, kaempferol, and ferulic acid through HPLC analysis, other active constituents may also be available in the crude extract that could contribute to the observed antiviral activity. Due to the complex nature of crude extracts and the relatively low abundance of individual phytochemical compounds in natural herbs, their bioactivity is often driven by synergistic or combinatorial interactions among multiple constituents rather than by individual compounds acting in isolation [86–90]. Notably, our HPLC analysis revealed an unidentified compound with a retention time of approximately 8.5 min, which showed high peak intensity, suggesting it represents an abundant constituent in ATE. The identity and contribution of other uncharacterized compounds that are responsible for ATE's antiviral activity remain to be elucidated, highlighting the need for comprehensive phytochemical profiling of this complex botanical extract.

In conclusion, we show here that ATE enhances antiviral defenses by stimulating the production of antiviral cytokines and activating key innate immune signaling pathways, including type I interferon and NF-κB signaling. This immune activation is evidenced by the upregulation of IFNs, pro-inflammatory cytokines, and phosphorylation of critical signaling molecules such as TBK1, IRF3, STAT1, and p65. These coordinated immunological responses contribute to the establishment of a robust antiviral state, effectively strengthening host defenses against a broad range of viral infections. After challenge with a lethal dose of Influenza viruses in mice, ATE showed reduced mortality due to reduced viral titers and less severe pathological changes in the lungs. Importantly, here we identified kaempferol, quercetin, and ferulic acid as the major bioactive compounds of ATE responsible for the observed antiviral and immunomodulatory properties. Taken together, *Aster tataricus* extract and its compounds may serve as promising natural antivirals or as lead candidates for drug design targeting viral infections in both humans and livestock.

## Supplementary Information

Supplementary material 1.

Supplementary material 2.

## Data Availability

The datasets analyzed during the current study are available from the corresponding author on reasonable request.

## Abbreviations

ATE: Aster tataricus extract
PR8: Influenza A (A/Puerto Rico/8/34) H1N1
HSV: Herpes Simplex Virus - type 1
NDV: Newcastle Disease Virus
GFP: Green fluorescent protein
HPLC: High performance liquid chromatography
IFN-I: Type I interferon
PRRs: Pattern recognition receptors
IFN-β: Interferon beta
rmIFN-β: Recombinant mouse Interferon beta protein+
IL-6: Interleukin 6
TNF-α: Tumor necrosis factor-α
IFN-γ: Interferon gamma
ELISA: Enzyme-linked immunosorbent assay
RT-PCR: Real time polymerase chain reaction
IFNAR: Interferon α/β receptor
TBK1: TANK-binding kinase 1
IRF3: Interferon regulatory factor 3
STAT1: Signal transducer and activator of transcription 1
NF-κB: Nuclear factor kappa B
IkBα: Nuclear factor of kappa light polypeptide gene enhancer in B-cells inhibitor, alpha
ISGs: Interferon-stimulated genes
PKR: Protein Kinase R
PML: Promyelocytic Leukemia Protein
GBP1: Guanylate-Binding Protein 1
OAS: 2′-5′-Oligoadenylate synthetase 1
MX1: Interferon-induced GTP-binding protein MX1
DW: Distilled water
PBS: Phosphate-buffered saline
RAW 264.7: Murine macrophages
Vero: African green monkey kidney cells
MDCK: Cocker spaniel kidney cells
DMEM: Dulbecco’s modified eagle’s medium
FBS: Fetal bovine serum
MOI: Multiplicity of infection
PFU: Plaque-forming unit
CC_50_: 50% cytotoxic concentration
EC _50_: 50% effective concentration
MTT: 3-(4,5-dimethylthiazol-2-yl)-2,5-diphenyltetrazolium bromide
hpi_50_: Hours post-infection
hpt: Hours post-treatment
dpi: Days post-infection
MLD_50_: 50% minimum lethal dose
TCID_50_: 50% tissue culture infectious dose
TPCK: L-1-tosylamide-2-phenylethyl chloromethyl ketone trypsin
CPE: Cytopathic effects
H&E: Hematoxylin and eosin
BALF: Bronchoalveolar lavage fluid
HBSS: Hank’s balanced salt solution
