# Supplementary material for: Aster tataricus extract and its active compounds display a broad spectrum of antiviral activity in vitro and in vivo
Source: Chin Med. 2025 Jul 15;20:113. doi: 10.1186/s13020-025-01167-1 (PMC12265312; doi:10.1186/s13020-025-01167-1)
Supplement: Supplementary file 1 — Supplementary material 1. [file 13020_2025_1167_MOESM1_ESM.docx]

***Aster tataricus* extract and its active compounds display a broad spectrum of antiviral activity *in vitro* and *in vivo***

**Supplementary Figures**

Nuwan Gamage^1†^, Ji-Won Cha^1†^, Ji-Soo Jeong^2^, Yebin Seong^1^, Kiramage Chathuranga^1^, Asela Weerawardhana^1^, Jin Yeul Ma^3^, Tae-Won Kim^2^, and Jong-Soo Lee^1^*

^1^Department of Microbiology, College of Veterinary Medicine, Chungnam National University, Daejeon, Republic of Korea

^2^Department of Pharmacology, College of Veterinary Medicine, Chungnam National University, Daejeon, Republic of Korea

^3^Korean Medicine Application Center, Korea Institute of Oriental Medicine, Daegu, Republic of Korea

Running Head: *Aster tataricus* Antiviral Activity *in vitro* and *in vivo*

†These authors have contributed equally to this work

*Address correspondence to: Jong-Soo Lee, jongsool@cnu.ac.kr

**
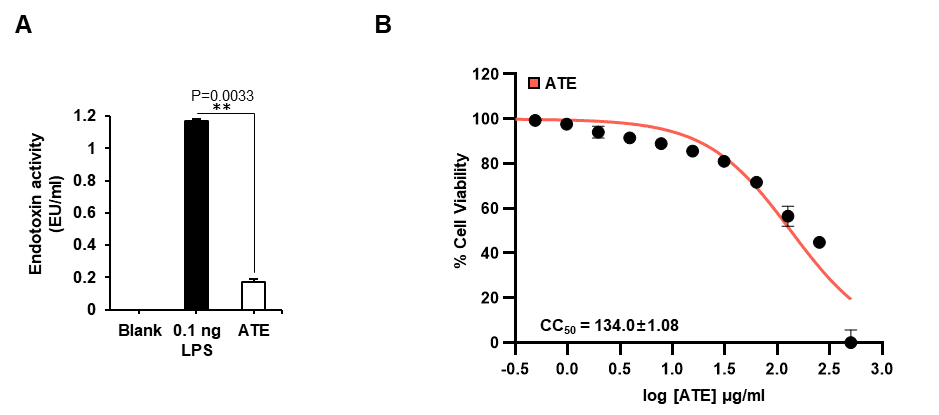
**

**Fig. S1** Cytotoxicity (CC_50_) Assessment and Endotoxin Testing of ATE. **(A)** ATE was tested for residual endotoxin contamination using a ToxinSensor™ Chromogenic LAL Endotoxin Assay Kit. **(B)** RAW264.7 cells were exposed to increasing concentrations of ATE, and cytotoxicity was assessed using the conventional MTT assay. Cell viabilities were expressed as a percentage. Values presented as mean ± SD. Error bars indicate the range of values obtained from counting duplicates in three independent experiments. (*P < 0.05, **P < 0.01, and ***P < 0.001 indicates a significant difference between groups).

**
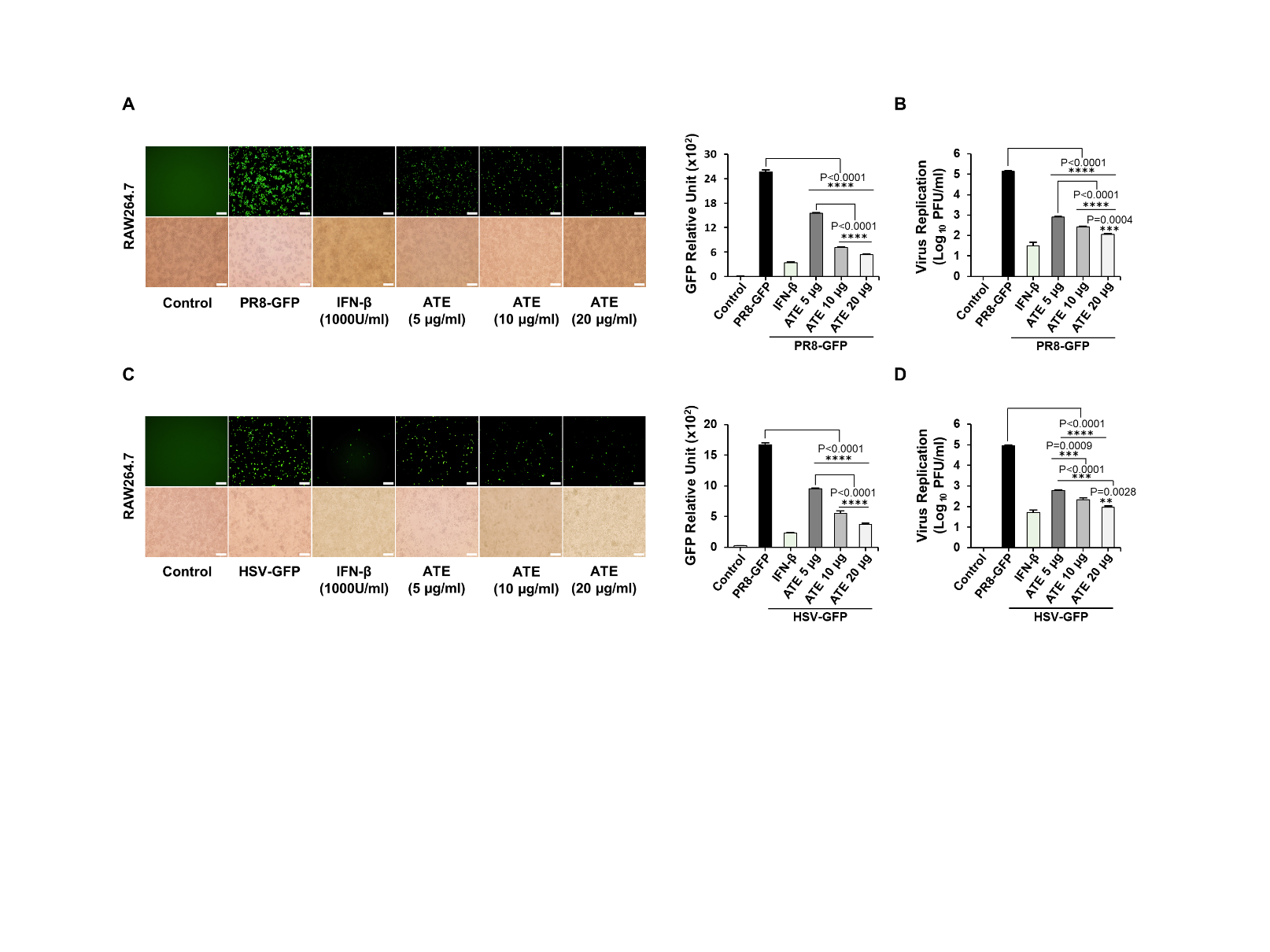
**

**Fig. S2** Pre-treatment with ATE inhibits replication of both RNA and DNA viruses in a concentration-dependent manner. RAW264.7 cells treated with ATE at 5, 10, or 20 μg/ml or 1,000 U/ml of rmIFN-β, 12 h before infection with **(A)** PR8-GFP, **(C)** HSV-GFP, at a 1 MOI, and 3 MOI, respectively. GFP fluorescence was measured at 24 hpi. Viruses were titrated from the infected cells using plaque assay **(B, D)**. Error bars indicate the range of values obtained from counting triplicates in three independent experiments. The scale bar represents 50 μm. (*P < 0.05, **P < 0.01, and ***P < 0.001 indicate a significant difference between groups).

**
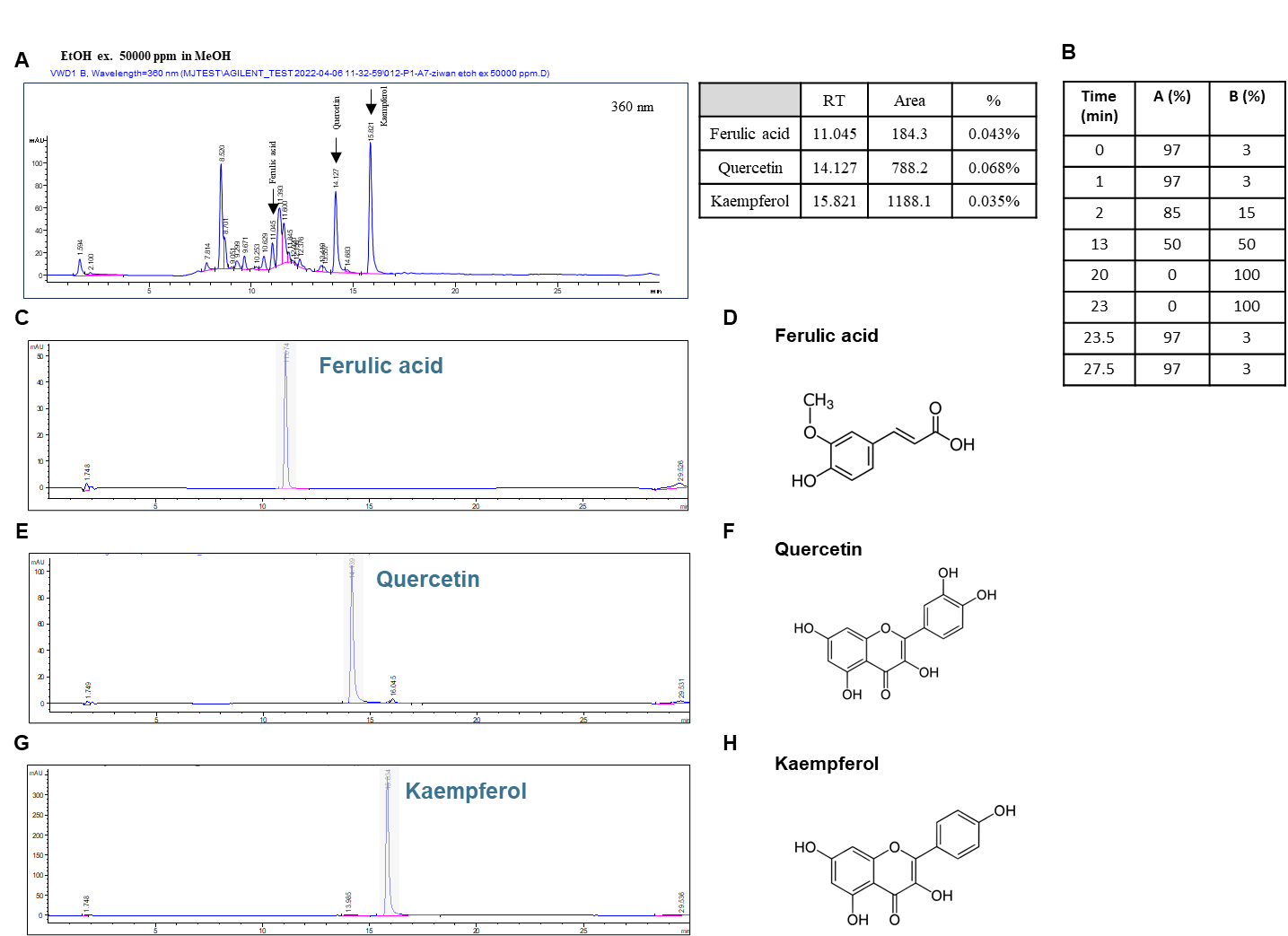
**

**Fig. S3** HPLC analysis. **(A)** HPLC was carried out to identify major active compounds present in ATE **(B)** HPLC analysis condition. The mobile phase consisted of 1 % Formic acid (Solvent A) and Acetonitrile (Solvent B) in the gradient mode as follows: 0–1 min 3 % B; 1–2 min 3–15 % B; 2–13 min 15–50 % B; 13–20 min 50–100 % B; 20–23 min 100 % B; 23–23.5 min 100–3 % B; 23.5–27.5 min 3 % B at a flow rate of 0.25 ml/min at 40 °C. Injection volume was 5 µl, and the UV detector was set at 360 nm. Quercetin, Kaempferol, and Ferulic acid were quantified in ATE. We picked up Ferulic acid **(C, D)**, Quercetin **(E, F)**, and Kaempferol **(G, H)** as major compounds present in ATE using a standard-based selection procedure.

**
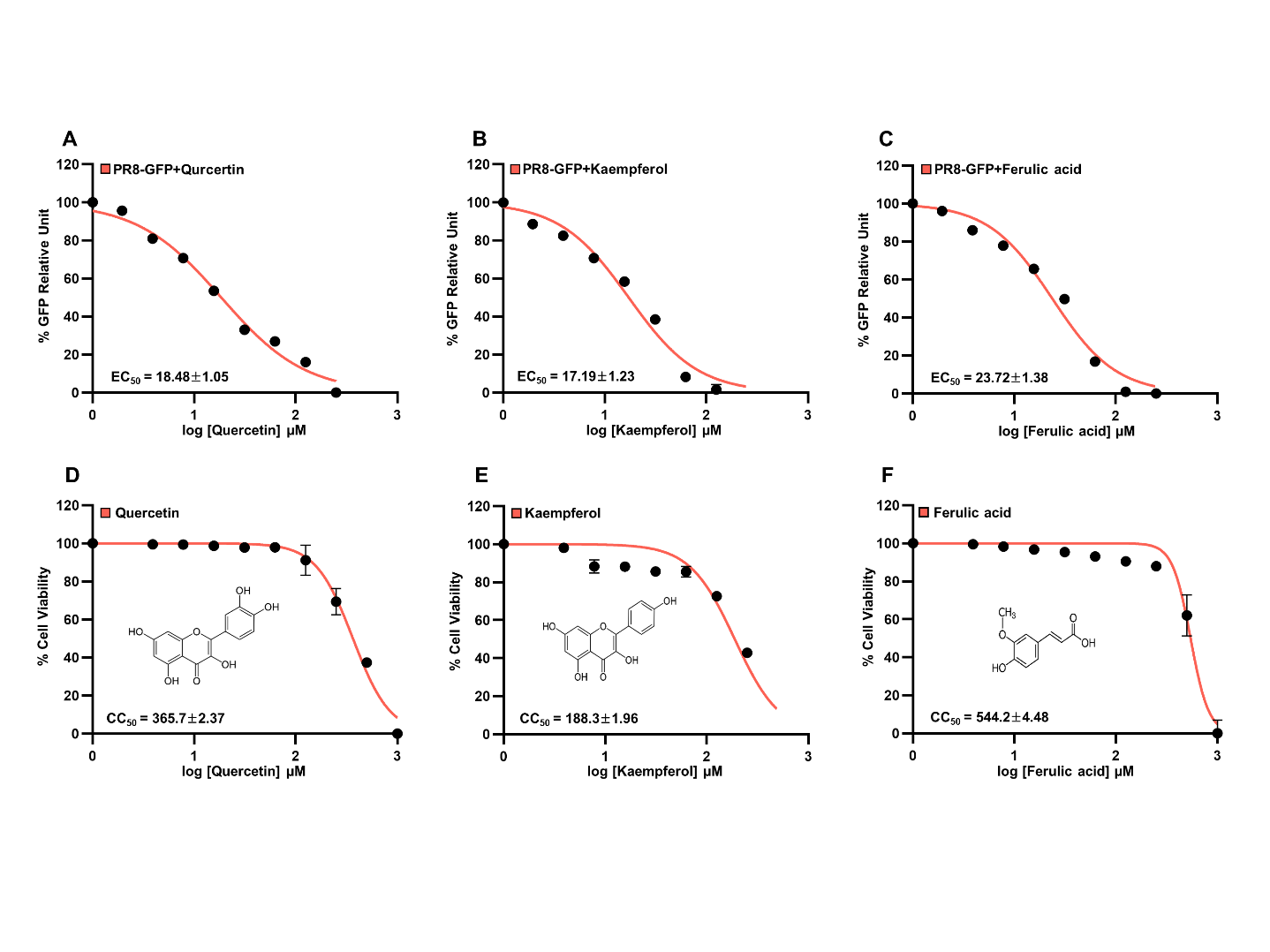
**

**Fig. S4.** Determination of EC_50_ and CC_50_ of active compounds identified in ATE. The 50% Effective concentration of **(A)** Quercetin, **(B)** Kaempferol, and **(C)** Ferulic acid was determined by GFP fluorescence reduction assay in RAW264.7 cells after *in vitro* infection with PR8-GFP. The 50% cytotoxic concentration of active compounds was determined by MTT assay **(D-F).** Percentage of GFP absorbance and cell viability are expressed as mean ± SD. Error bars indicate the range of values obtained from counting duplicates in three independent experiments. (*P < 0.05, **P < 0.01, and ***P < 0.001 indicates a significant difference between groups).
