# Supplementary material for: Aster tataricus extract and its active compounds display a broad spectrum of antiviral activity in vitro and in vivo
Source: Chin Med. 2025 Jul 15;20:113. doi: 10.1186/s13020-025-01167-1 (PMC12265312; doi:10.1186/s13020-025-01167-1)
Supplement: Supplementary file 2 — Supplementary material 2. [file 13020_2025_1167_MOESM2_ESM.docx]

***Aster tataricus* extract and its active compounds display a broad spectrum of antiviral activity *in vitro* and *in vivo***

**Supplementary Tables**

Nuwan Gamage^1†^, Ji-Won Cha^1†^, Ji-Soo Jeong^2^, Yebin Seong^1^, Kiramage Chathuranga^1^, Asela Weerawardhana^1^, Jin Yeul Ma^3^, Tae-Won Kim^2^, and Jong-Soo Lee^1^*

^1^Department of Microbiology, College of Veterinary Medicine, Chungnam National University, Daejeon, Republic of Korea

^2^Department of Pharmacology, College of Veterinary Medicine, Chungnam National University, Daejeon, Republic of Korea

^3^Korean Medicine Application Center, Korea Institute of Oriental Medicine, Daegu, Republic of Korea

Running Head: *Aster tataricus* Antiviral Activity *in vitro* and *in vivo*

†These authors have contributed equally to this work

*Address correspondence to: Jong-Soo Lee, jongsool@cnu.ac.kr

**Table S1: List of mouse primers used for real-time PCR**

**
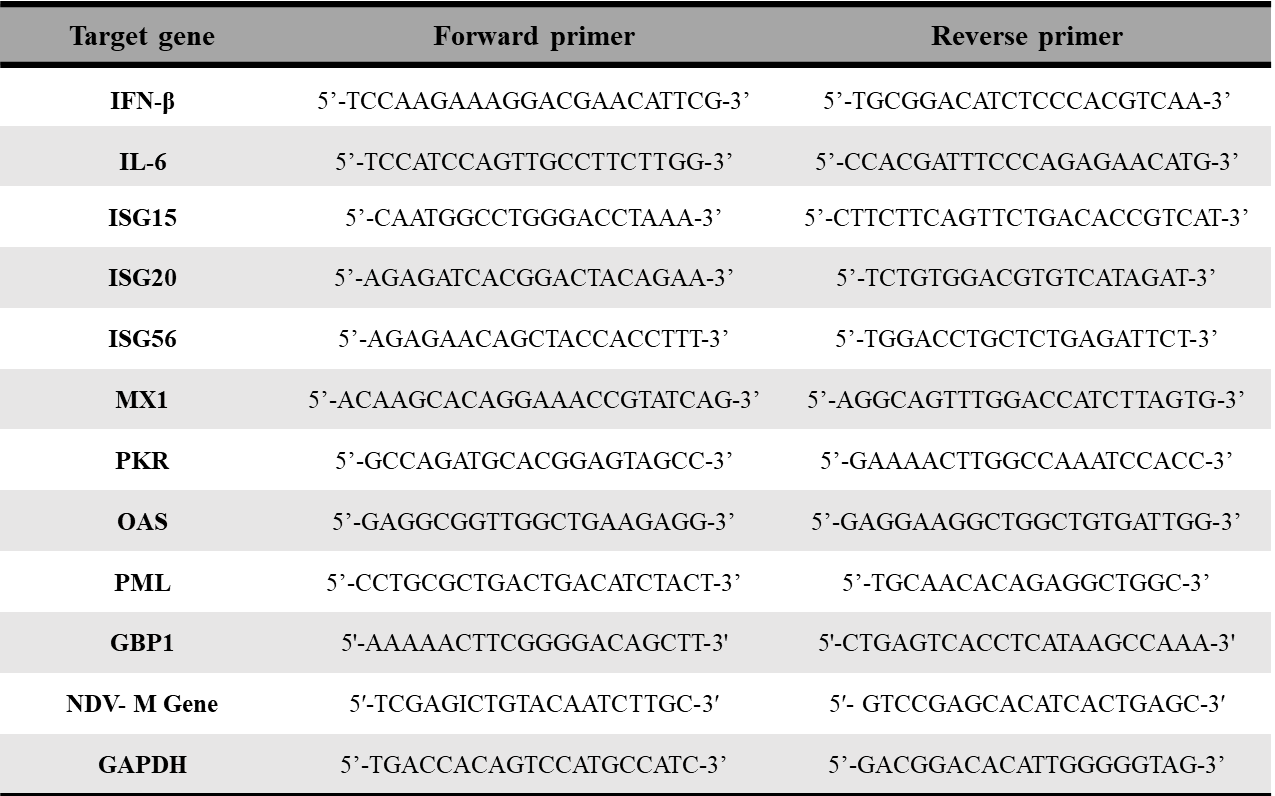
**

**Table S2: *In vivo* (mouse) experiments against influenza A virus infectio**

**
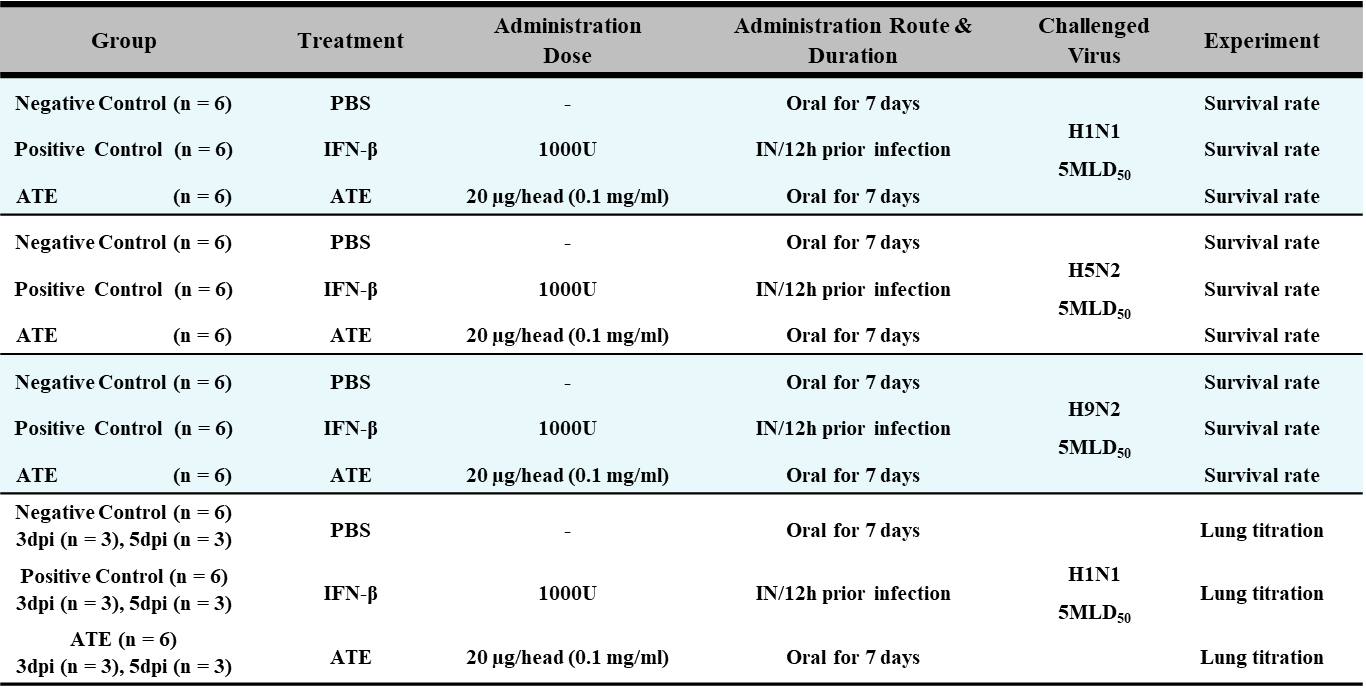
**
